# Persistent osteoarticular pain in children: early clinical and laboratory findings suggestive of acute lymphoblastic leukemia (a multicenter case-control study of 147 patients)

**DOI:** 10.1186/s12969-019-0376-8

**Published:** 2020-01-02

**Authors:** Mathilde Louvigné, Josué Rakotonjanahary, Laurence Goumy, Aude Tavenard, Jean-François Brasme, Fanny Rialland, André Baruchel, Marie-Françoise Auclerc, Véronique Despert, Marie Desgranges, Sylvie Jean, Albert Faye, Ulrich Meinzer, Mathie Lorrot, Chantal Job-Deslandre, Brigitte Bader-Meunier, Virginie Gandemer, Isabelle Pellier, on behalf of the GOCE Group, Emilie De Carli, Stéphanie Proust, Coralie Mallebranche, Mylène Duplan, Chloé Henry, Liana Carausu, Sophie Haro, Fanny Falaque, Damien Bodet, Marianna Deparis, Caroline Thomas, Sara Weinhard, Marie-Laure Couec, Estelle Thébaud, Morgane Cleirec, Frédéric Millot, Laurence Blanc, Chrystelle Dupraz, Natacha Maledon, Jacinthe Bonneau, Céline Chappe, Sophie Taque, Fabienne Toutain, Sophie Pertuisel, Chloé Puiseux, Jeremie Gaudichon, Pascale Blouin, Marion Gillibert Yvert, Anne Jourdain, Charlotte Hucault, Jill Serre, Julien Lejeune

**Affiliations:** 1 Unité d’Onco-Hémato-Immunologie pédiatrique, CHU Angers, 4 rue Larrey, 49933 Angers, France; 2 Service de Pédiatrie générale, CHU Angers, Angers, France; 3 Unité d’Onco-Hémato-Immunologie pédiatrique, CHU Rennes, Rennes, France; 4 Unité d’Onco-Hémato-Immunologie pédiatrique, CHU Nantes, Nantes, France; 5 Unité d’Hémato-Immunologie pédiatrique, CHU Robert Debré, Hôpitaux de Paris, Paris, France; 6 Université de Paris, UFR Paris Diderot, Paris, France; 7 Service de Pédiatrie générale, CHU Rennes, Rennes, France; 8 Service de Pédiatrie générale Maladies Infectieuses et Médecine Interne, Centre de référence des rhumatismes inflammatoires et maladies auto-immunes systémiques rares de l’enfant RAISE, CHU Robert Debré, Hôpitaux de Paris, Paris, France; 9 Unité d’Immuno-Hématologie et Rhumatologie Pédiatriques, Hôpital Necker-Enfants Malades, Hôpitaux de Paris, Paris, France

**Keywords:** Childhood acute lymphoblastic leukemia, Juvenile idiopathic arthritis, Diagnosis, Bone pain, Arthralgia

## Abstract

**Background:**

The aim of this study was to identify early clinical and laboratory features that distinguish acute lymphoblastic leukemia (ALL) from juvenile idiopathic arthritis (JIA) in children presenting with persistent bone or joint pain for at least 1 month.

**Methods:**

We performed a multicenter case-control study and reviewed medical records of children who initially presented with bone or joint pain lasting for at least 1 month, all of whom were given a secondary diagnosis of JIA or ALL, in four French University Hospitals. Each patient with ALL was paired by age with two children with JIA. Logistic regression was used to compare clinical and laboratory data from the two groups.

**Results:**

Forty-nine children with ALL and 98 with JIA were included. The single most important feature distinguishing ALL from JIA was the presence of hepatomegaly, splenomegaly or lymphadenopathy; at least one of these manifestations was present in 37 cases with ALL, but only in 2 controls with JIA, for an odds ratio (OR) of 154 [95%CI: 30–793] (regression coefficient: 5.0). If the presence of these findings is missed or disregarded, multivariate analyses showed that non-articular bone pain and/or general symptoms (asthenia, anorexia or weight loss) (regression coefficient: 4.8, OR 124 [95%CI: 11.4–236]), neutrophils < 2 × 10^9^/L (regression coefficient: 3.9, OR 50 [95%CI: 4.3–58]), and platelets < 300 × 10^9^/L (regression coefficient: 2.6, OR 14 [95%CI: 2.3–83.9]) were associated with the presence of ALL (area under the ROC curve: 0.96 [95%CI: 0.93–0.99]).

**Conclusions:**

Based on our findings we propose the following preliminary decision tree to be tested in prospective studies: in children presenting with at least 1 month of osteoarticular pain and no obvious ALL in peripheral smear, perform a bone marrow examination if hepatomegaly, splenomegaly or lymphadenopathy is present. If these manifestations are absent, perform a bone marrow examination if there is fever or elevated inflammatory markers associated with non-articular bone pain, general symptoms (asthenia, anorexia or weight loss), neutrophils < 2 × 10^9^/L or platelets < 300 × 10^9^/L.

## Background

Acute lymphoblastic leukemia (ALL) is the most common childhood malignancy, accounting for 25% of cancers in children [1, 2]. In 15–30% of cases, ALL is diagnosed because of isolated and persistent osteoarticular complaints without clear laboratory features which could direct the diagnosis (normal blood count and absence of blasts in peripheral blood smear) [3]. Moreover, this form of ALL is associated with a lower incidence of hepatomegaly, splenomegaly or lymphadenopathy [4]. The time between the first episode of bone or joint pain and a bone marrow examination is sometimes a period of several months [5, 6] and there can be a long succession of visits to either the orthopedist or the rheumatologist pediatrician [7]. The first diagnosis, proposed for 76.5% of cases of bone and articular manifestations, is juvenile idiopathic arthritis (JIA) [5], the most common rheumatic disease in children [8, 9], defined according to the International League of Associations for Rheumatology (ILAR) criteria [10]. However, this is a diagnosis of exclusion that rules out other rheumatic or infectious diseases and malignancies, such as ALL [10]. ALL and JIA can both present with chronic joint and periarticular complaints, fever, hepatosplenomegaly or lymphadenopathy, anemia and elevated inflammatory markers [11, 12]. A bone marrow examination is the best way for the clinician to diagnose ALL, especially when blasts are absent in peripheral blood smear [13]. When there is no hepatosplenomegaly or lymphadenopathy, and when osteoarticular complaints are localized, it seems to be harder to distinguish ALL from non-systemic forms of JIA.

Some studies have compared clinical and laboratory data obtained before the diagnosis of ALL or JIA in children presenting with chronic osteoarticular manifestations. In the case of ALL, most of them described sudden, intense and localized periarticular pain, whereas in the case of JIA, articular complaints appear in a more progressive manner, with morning stiffness and arthritis [11, 14–16]. With regard to laboratory data, it has been described that in the event of ALL, platelets and leukocytes are normal or decreased, whereas their rate is often elevated in the case of JIA [11, 12, 14]. Nevertheless, these different parameters seem to be insufficient for clinicians to distinguish ALL from JIA.

The aim of this study was to describe early clinical and laboratory features associated with ALL in children presenting with isolated persistent osteoarticular pain for at least one month. These criteria could efficiently lead to the diagnosis of ALL rather than JIA, and allow physicians to perform a bone marrow examination as early as possible, before the appearance of hepatomegaly, splenomegaly or lymphadenopathy, high lymphocyte levels, cytopenia and blasts in peripheral blood smear [3].

## Methods

This is a multicenter case-control study of children presenting with at least 1 month of persistent bone or joint pain and who were ultimately diagnosed with ALL or JIA. In this study we included children under the age of 18 who were diagnosed with ALL on the basis of bone marrow examination results, after at least 1 month of persistent bone or joint pain without associated pancytopenia or blasts in peripheral blood smear at the beginning of complaints, between October 1, 2000 and March 31, 2011, in the French University Hospitals of Angers, Nantes, Rennes and Robert Debré (Paris). All patients were included in the French national protocol FRALLE 2000. Pancytopenia was defined as the association of neutrophils, hemoglobin and platelets below the normal values for the age and gender. Each patient was paired by age with two children with JIA, followed up at Rennes, Angers and Robert Debré University Hospitals (competence centers for pediatric rheumatological diseases), who presented with at least 1 month of isolated bone or joint pain, and who were diagnosed between September 1, 1998 and November 31, 2014. JIA was diagnosed according to the ILAR criteria defined in 2001 [10]. All JIA children were within 2 years of age of their respective ALL patients.

For each child, we identified information pertaining to their demographic profile, symptoms and clinical manifestations at diagnosis and laboratory investigations carried out since the onset of pain was recorded. The term “localized initial presentation” was used if only one bone or joint was painful at the onset of symptoms. In other cases, we considered the initial presentation as “diffuse”. “Time to diagnosis” was defined as the interval between the first symptoms and diagnosis. “General symptoms” were defined as the presence of at least one of the following parameters: anorexia, weight loss or asthenia. “Weight loss” was defined as a weight decrease of more than 5%. “Elevated inflammatory markers” were defined as C-reactive protein (CRP) > 6 mg/L and/or the first hour’s erythrocyte sedimentation rate (ESR) > 20 mm. For each child, we calculated the mean and median values of all laboratory data collected prior to the diagnosis. The study was approved by the ethics committee of Angers University Hospital (a signed consent form was not required prior to patients’ inclusion because of the retrospective character of the study and the non-nominal data collection). The reporting of the study was performed according to TRIPOD recommendations [17].

### Statistical analysis

Descriptive statistics were used to summarize the characteristics of children with ALL and children with JIA. Variables related to disease and laboratory, demographic, and clinical characteristics were analyzed and described. A multivariate analysis was performed using a logistic regression model to study factors associated with ALL in children with persistent bone or joint pain. Two analyses with two separate models were performed. In the first one, the following factors were considered as possible variables: gender, non-articular bone pain, joint pain, general symptoms, arthritis, fever and the presence of at least one of the following manifestations: hepatomegaly, splenomegaly or lymphadenopathy. We also included laboratory tests results collected prior to the diagnosis: white blood cell (WBC) count, neutrophils, lymphocytes, hemoglobin (Hgb), platelets, mean corpuscular volume (MCV), ESR and CRP. In the second model, data related to “hepatomegaly, splenomegaly or lymphadenopathy” were excluded as possible variables. For the logistic regression, a step-down variable selection using the Akaike Information Criterion (AIC) was used as a stopping rule [18–20].

Analyses of the whole sample were performed using the MICE procedure (Multivariate Imputation by Chained Equations) for the management of missing data. We assumed data were missing at random. The MICE procedure was used in Stata software.

The area under the ROC curve (AUC) was described for each final model. An AUC ≥ 75% was considered acceptable. All the statistical tests were performed under a one-sided significance level of 0.05. The statistical analysis was completed using Stata software 12.1 (StataCorp. Texas).

## Results

We included 49 patients with a diagnosis of ALL and 98 with a diagnosis of JIA. The most frequent forms of JIA in our study were oligoarthritis, polyarthritis and enthesitis-related arthritis, which accounted for 43.9, 29.6 and 18.4% of control patients, respectively. Of the JIA patients, 5.1% suffered from systemic arthritis and 3.1% suffered from psoriatic arthritis. All patients with ALL had a B-form (B-precursor ALL), although it was not a selection criterion for the inclusion in the study. Nine of the ALL patients were followed by a rheumatologist and four of them had been treated for oligoarticular or polyarticular JIA before diagnosis of ALL, whereas only one had arthritis at diagnosis of ALL.

The demographical and clinical characteristics of these patients are described in Table 1. The gender ratio was in favor of boys in the ALL group and girls in the JIA group. The median time to diagnosis was two times shorter for patients with ALL (Table 1). Non-articular bone pain was more frequent for children presenting with ALL, whereas joint pain was described in high rates in the case of both pathologies. As for clinical examinations at diagnosis, only one patient with ALL had arthritis and only two patients in the JIA group had “hepatomegaly, splenomegaly or lymphadenopathy” (one had enthesitis-related arthritis and the other one had systemic JIA) (Table 1). Eighteen patients with JIA had no arthritis at the time of the physical exam made by pediatricians or rheumatologists experienced in pediatric rheumatology. The diagnosis was made because these patients had a medical history consistent with JIA even if arthritis had been resolved at diagnosis.

**Table 1:** Demographical and clinical characteristics of patients with ALL or JIA

	ALL	JIA	*p*
N	49	98	
Age, y (median [interquartile range])	7.3 [3.6–12.4]	7.6 [3.1–12.4]	NS**
Sex ratio, males per female	1.9	0.7	< 0.01*
Time to diagnosis, days (median [interquartile range])	57 [38;90]	121 [69;266]	< 0.001**
Pain location, n (%)			
Joint pain	40 (82)	98 (100)	NS*
Non-articular pain	18 (37)	7 (7)	< 0.001*
Initial presentation, n (%)			< 0.05*
Diffuse	42 (86)	68 (69)	
Localized	7 (14)	30 (31)	
Symptoms at diagnosis, n (%)			
Fever	30 (61)	12 (12)	< 0.001*
General symptoms			
Asthenia	34 (69)	7 (7)	< 0.001*
Anorexia	12 (24)	4 (4)	< 0.001*
Weight loss	10 (20)	4 (4)	< 0.01***
Clinical manifestations at diagnosis, n (%)			
Arthritis	1 (2)	80 (82)	< 0.001*
Hepatomegaly, splenomegaly or lymphadenopathy	37 (76)	2 (2)	< 0.001*
Hepatomegaly	23 (47)	1 (1)	< 0.001*
Splenomegaly	15 (31)	1 (1)	< 0.001*
Lymphadenopathy	28 (57)	1 (1)	< 0.001*
Anemia signs	25 (51)	1 (1)	< 0.001*
Thrombocytopenia signs	10 (20)	0 (0)	< 0.001***
Neurological disorders	8 (16)	2 (2)	NS***
Dermatological signs	1 (2)	7 (7)	NS***

Univariate analysis with χ^2^ (*), Wilcoxon (**) or Fisher (***) tests

*ALL* Acute lymphoblastic leukemia, *JIA* Juvenile idiopathic arthritis, *NS* Not significant

“Time to diagnosis” was defined as the interval between the first symptoms and diagnosis

The term “localized initial presentation” was used if only one bone or joint was painful at the onset of symptoms. In other cases, we considered the initial presentation as “diffuse”

Before the diagnosis was carried out, there were no differences in the lymphocyte rate between the two groups. Neutrophils, eosinophils, basophils, monocytes, platelets, Hgb and hematocrit (HCT) were lower for ALL patients (*p* ≤ 0.001). We found a higher MCV for ALL children (*p* = 0.009) (Table 2). In cases of ALL, CRP and ESR were higher (Table 2). The same trends were observed at the time of final diagnosis except for lymphocytes which were lower in the ALL group compared to JIA patients (Table 3). We observed moderate anemia, neutropenia and thrombocytopenia, without elevation of lymphocytes and leukocytes for the ALL group (Table 3). In the small subgroup of systemic JIA, we observed elevated neutrophil counts (median value: 12.7 [IQR: 11.5–20.6] × 10^9^/L) and platelet counts (median value: 506 [IQR: 401–512] × 10^9^/L). Bone marrow examination was undertaken for all patients of the ALL group and for one patient with JIA.

**Table 2:** Laboratory tests results collected before the diagnosis in ALL and JIA patients

	ALL	JIA	p*
Blood count	(median [interquartile range])	(median [interquartile range])	
WBC count, 10^9^/L	5.1 [4.2–6.5]	8.4 [7.0–10.3]	< 0.001
Neutrophils, 10^9^/L	1.6 [0.8–2.9]	4.6 [3.3–5.4]	< 0.001
Eosinophils, 10^9^/L	0.02 [0–0.06]	0.2 [0.1–0.3]	< 0.001
Basophils, 10^9^/L	0.003 [0–0.03]	0.03 [0.02–0.05]	< 0.001
Lymphocytes, 10^9^/L	2.6 [1.8–4.2]	2.9 [2.3–3.6]	0.56
Monocytes, 10^9^/L	0.2 [0.1–0.4]	0.6 [0.5–0.8]	< 0.001
Hgb, g/dL	10.6 [9.2–12.1]	11.9 [11,3-12,9]	0,001
HCT, %	31.8 [27.0–35.5]	35.9 [33.3–38.6]	< 0.001
MCV, fL	83 [78–86]	80 [76–83]	0.009
MCHC, g/dL	34 [33–34]	34 [33–34]	0.84
Platelets, 10^9^/L	218 [158–310]	377 [298–439]	< 0.001
Inflammatory parameters			
1st hr. ESR, mm	56 [35–98]	25 [12–48]	< 0.001
CRP, mg/L	46 [9–80]	8 [2–26]	< 0.001

* Univariate analysis with Wilcoxon test

*ALL* Acute lymphoblastic leukemia, *CRP* C-reactive protein, *ESR* Erythrocyte sedimentation rate, *HCT* Hematocrit, *Hgb* Hemoglobin, *JIA* Juvenile idiopathic arthritis, *MCHC* Mean corpuscular hemoglobin concentration, *MCV* Mean corpuscular volume, *WBC* White blood cells

**Table 3:** Laboratory tests results collected at diagnosis in ALL and JIA patients

	ALL	JIA	p*
	(median [interquartile range])	(median [interquartile range])	
Blood count			
WBC count, 10^9^/L	5.3 [3.0–9.5]	8.3 [6.6–10.1]	< 0.001
Neutrophils, 10^9^/L	0.8 [0.5–1.9]	4.0 [3.0–5.7]	< 0.001
Eosinophils, 10^9^/L	0.03 [0–0.08]	0.02 [0.14–0.33]	< 0.001
Basophils, 10^9^/L	0 [0–0]	0.03 [0.02–0.05]	< 0.001
Lymphocytes, 10^9^/L	2.2 [1.8–3.4]	3.0 [2.2–3.8]	0.02
Monocytes, 10^9^/L	0.1 [0.05–0.3]	0.6 [0.5–0.8]	< 0.001
Hgb, g/dL	8.8 [7.5–10.6]	11.9 [11.1–12.6]	< 0.001
HCT, %	26.0 [22.5–30.1]	35.7 [33.7–37.6]	< 0.001
MCV, fL	83 [78–86]	80 [76–83]	0.01
MCHC, g/dL	34 [33–34]	34 [33–34]	0.64
Platelets, 10^9^/L	121 [71–213]	357 [295–430]	< 0.001
Inflammatory parameters			
1st hr. ESR, mm	75 [36–106]	18 [7–55]	< 0.001
CRP, mg/L	32 [12–77]	3 [0–24]	< 0.001

* Univariate analysis with Wilcoxon test

*ALL* Acute lymphoblastic leukemia, *CRP* C-reactive protein, *ESR* Erythrocyte sedimentation rate, *HCT* Hematocrit, *Hgb* Hemoglobin, *JIA* Juvenile idiopathic arthritis, *MCHC* Mean corpuscular hemoglobin concentration, *MCV* Mean corpuscular volume, *WBC* White blood cells

In the multivariate analysis, the following factors were significantly associated with ALL: the presence of hepatomegaly, splenomegaly or lymphadenopathy (regression coefficient: 5.0, OR 154, 95%CI: 30–793, *p* <  0.001), non-articular bone pain (regression coefficient: 2.6, OR 13.6, 95%CI: 3.2–57.4, *p* = 0.001) and fever (regression coefficient: 1.5, OR 4.5, 95%CI: 1.9–10.5, *p* = 0.01) (Table 4). When performing a multivariate analysis excluding data relative to “hepatomegaly, splenomegaly or lymphadenopathy”, the following factors were associated with ALL: non-articular bone pain and/or general symptoms (asthenia, anorexia and weight loss) (regression coefficient: 4.8, OR 124, 95%CI: 11.4–236, *p* <  0.001), neutrophil counts < 2 × 10^9^/L (regression coefficient: 3.9, OR 50, 95%CI: 4.3–58, *p* = 0.002) and platelet counts < 300 × 10^9^/L (regression coefficient: 2.6, OR 14, 95%CI: 2.3–83.9, *p* = 0.004) (Table 4). We then separately analyzed 92 children who presented with fever and/or elevated inflammatory markers: 38 with ALL and 54 with JIA. The same factors were associated with ALL: non-articular bone pain and/or general symptoms (regression coefficient: 4.6, OR 104, 95%CI: 6.3–172, *p* = 0.001), neutrophil counts < 2 × 10^9^/L (regression coefficient: 4.6, OR 104, 95%CI: 3.4–327, *p* = 0.008) and platelet counts < 300 × 10^9^/L (regression coefficient: 3.4, OR 31.8, 95%CI: 2.9–353, *p* = 0.005) with higher levels of significance (Table 4). The model performance measures (AUC, sensitivity and specificity) showed acceptable measures (Table 4).

**Table 4:** Factors associated with ALL (Univariate and Multivariate analyses)

	_____Univariate analysis______			___Multivariate analysis 1^a^_____			___Multivariate analysis 2^b^_____			___Multivariate analysis 3^c^_____		
	β	OR (95% CI)	*p*	β	OR (95% CI)	*p*	β	OR (95% CI)	*p*	β	OR (95% CI)	*p*
- Non-articular bone pain	2.0	7.5 (2.9–19.8)	< 0.001	2.6	13.6 (3.2–57.4)	0.001	–	–	–	–	–	–
- Non-articular bone pain and/or poor general state	3.5	33.2 (12.5–87.6)	< 0.001	–	–	–	4.8	124 (11.4–236)	< 0.001	4.6	104 (6.3–172)	0.001
- Neutrophils < 2 × 10^9^/L	1.8	6.1 (2.3–16.1)	< 0.001	–	–	–	3.9	50 (4.3–58)	0.002	4.6	104 (3.4–327)	0.008
- Platelets < 300 × 10^9^/L	2.0	7.6 (2.3–19.8)	< 0.001	–	–	–	2.6	14 (2.3–83.9)	0.004	3.4	31.8 (2.9–353)	0.005
- Hepatomegaly, splenomegaly or lymphadenopathy	4.9	148 (31–696)	< 0.001	5.0	154 (30–793)	< 0.001	–	–	–	–	–	–
- Fever	1.3	3.6 (2.1–6.5)	< 0.001	1.5	4.5 (1.9–10.5)	0.01	–	–	–	–	–	–
Model performance measures ^d^												
- AUC (95% CI)					0.94 (0.89–0.98)			0.96 (0.93–0.99)			0.96 (0.93–0.99)	
- Sensitivity (95% CI)					0.85 (0.81–0.90)			0.83 (0.80–0.88)			0.83 (0.79–0.99)	
- Specificity (95% CI)					0.93 (0.89–0.98)			0.95 (0.91–0.99)			0.95 (0.92–0.99)	

β is the regression coefficient

^a^: Multivariate analysis including “hepatomegaly, splenomegaly or lymphadenopathy”

^b^: Multivariate analysis after excluding “hepatomegaly, splenomegaly or lymphadenopathy”

^c^: Multivariate analysis after excluding “hepatomegaly, splenomegaly or lymphadenopathy”: model restricted to children with fever and/or increased inflammatory markers

^d^: Values of apparent model performance obtained in the same subjects used for model development (AUC: area under the ROC curve)

At the onset of symptoms, radiographies were performed on 30 children in the ALL group and 84 with JIA. They were considered normal in 23 children with ALL and 52 with JIA. In the ALL group, three children had osteolytic lesions, one had radiolucent metaphyseal bands, two had a periosteal reaction and one had bone-marrow infiltration. In the JIA group, 17 children had joint effusion, 16 had soft tissue swelling, four had epiphyseal lesions, two had osteochondritis and two had joint space narrowing. Ultrasonography was performed in seven patients with ALL and was normal for all seven. For the JIA group, at the beginning of complaints, ultrasonography was normal for 10 patients and identified joint effusion in 30 patients and synovitis in 18 patients. A scintigraphy, which was performed for nine patients with ALL and 19 with JIA, found abnormal radionuclide uptake in eight children with ALL and 12 with JIA. In the ALL group, eight patients had an MRI and two had a CT-scan, which identified bone-marrow infiltration and osteolysis. In the JIA group, 24 children had an MRI and five had a CT-scan, which mostly found synovial hypertrophy and joint effusion.

## Discussion

In this study, we described early parameters that could help clinicians to distinguish ALL from JIA in children with persistent bone or joint pain.

First, we observed that the presence of hepatomegaly, splenomegaly or lymphadenopathy is clearly associated with an ALL diagnosis in cases of chronic osteoarticular complaints in children**.** Only two patients in the JIA group had hepatomegaly, splenomegaly or lymphadenopathy at diagnosis. This could be explained by the low prevalence of systemic JIA (5.1% vs 10–20% in the literature [9, 21]). This form usually presents from the outset with the association of fever and osteoarticular pain. They could therefore not be included in our study, which focused on children with isolated osteoarticular complaints. Tamashiro et al. observed that hepatomegaly was more frequent with a diagnosis of ALL rather than systemic JIA (54% vs 32%, *p* = 0.0075) in children with musculoskeletal symptoms. The difference was not significant for splenomegaly, and lymphadenopathy was more frequent in systemic JIA in their study (46% vs 21%, *p* = 0.002) [11]. Therefore, clinicians must examine bone marrow if a child presents with persistent bone or joint pain associated with hepatomegaly, splenomegaly or lymphadenopathy, even if systemic arthritis is considered.

When hepatomegaly, splenomegaly or lymphadenopathy are absent, it may be difficult to diagnose ALL. We observed that fever, general symptoms (asthenia, anorexia or weight loss) and non-articular bone pain were most frequently found in ALL rather than in JIA in the event of chronic osteoarticular complaints. Tamashiro et al. also found that weight loss was more frequent in ALL than in systemic JIA (30 vs 8%, *p* = 0.0005) [11]. Like us, Cabral and Tucker, Tamashiro et al. and Gupta et al. noted that intense metaphyseal bone pain, which occurs with a sudden onset, was a manifestation that pointed towards ALL, whereas JIA cases presented with more progressive joint pain associated with arthritis and morning stiffness [7, 11, 14]. Only one patient with ALL had arthritis in our study. As such, clinicians must ask about the occurrence of general symptoms and obtain a precise description of the location of the pain when they examine children with chronic osteoarticular complaints. We also encourage pediatricians or clinicians not specialized in rheumatology to search for arthritis when performing the clinical examination and ultrasonography. Indeed, when a child presents with multiple osteoarticular complaints, pediatricians might not have thought to perform an ultrasound. Ultrasonography has many advantages over other imaging techniques as it is easily accessible, fast, dynamic, avoids exposing the patient to radiation, does not require sedation, and has been shown to be superior to clinical examination in the detection of synovitis in JIA [22, 23]. We encourage clinicians to examine bone marrow in cases of non-articular bone pain without arthritis, or if osteoarticular complaints are associated with fever or general symptoms such asthenia, anorexia or weight loss.

In terms of laboratory features, lower neutrophil and platelet levels were associated with ALL, and this was more important and significant for children who presented with fever and/or elevated inflammatory markers. We chose to focus on this group of children because fever and/or elevated inflammatory markers are commonly found in cases of ALL or JIA and are consequently not used to distinguish between these two pathologies. When hepatomegaly, splenomegaly, lymphadenopathy or general symptoms such asthenia, anorexia and weight loss are absent, other factors should be considered to help the decision-making process, and we recommend that clinicians carefully analyze the blood count. The discordance between low or normal neutrophil or platelet counts and elevated inflammatory markers or fever, suggesting relative bone marrow failure, should lead to a bone marrow examination. This was previously observed by Trapani et al. and Cabral and Tucker [7, 16]. Tamashiro et al. also noted that neutrophil levels were less elevated in ALL than in systemic JIA [11]. Indeed, in the small subgroup of systemic JIA, we noted the same trend with an increase in neutrophil counts (median value: 12.7 [IQR: 11.5–20.6] × 10^9^/L) and platelet counts (median value: 506 [IQR: 401–512] × 10^9^/L). Jones et al. found that a low-normal platelet count (between 150 and 200 × 10^9^/L) was a sensitive and specific factor for the diagnosis of ALL, in the event of chronic osteoarticular complaints (sensitivity: 82% and specificity: 87%) [12]. We therefore suggest that clinicians examine bone marrow if a child presents with persistent bone or joint pain associated with fever or elevated inflammatory markers and neutrophils < 2 × 10^9^/L or platelets < 300 × 10^9^/L. We also encourage them to repeat blood counts when osteoarticular pain persists.

The retrospective analysis of the data is one of the limitations of our study. Biological samples were consequently not protocolized and we could not analyze some interesting data (e.g. lactate dehydrogenase, aspartate transaminase, auto-immunity data). Additionally, the imaging data could not be analyzed because of the absence of a centralized review. A prospective study, including more patients and especially more children with systemic JIA, is necessary in order to confirm our findings. Additionally, our study would have been more performant with a greater sample size, permitting one to perform data splitting and proceed to an internal validation of the different models according to TRIPOD recommendations [17, 24]. However, we observed acceptable values of model performances (AUC, sensitivity, specificity – Table 4) and as our study population was recruited from several centers, we assumed our samples were quite representative of children with ALL and JIA. Another limitation of our study is the definition of the control group. The primary focus of this study was to describe parameters which can differentiate ALL from JIA in children. However, it would be interesting for clinicians to consider all children with chronic osteoarticular pain who may be suffering from ALL, JIA or even other pathologies such as viral synovitis, bacterial arthritis, osteomyelitis, tendinitis and other benign diseases.

**Fig. 1:**
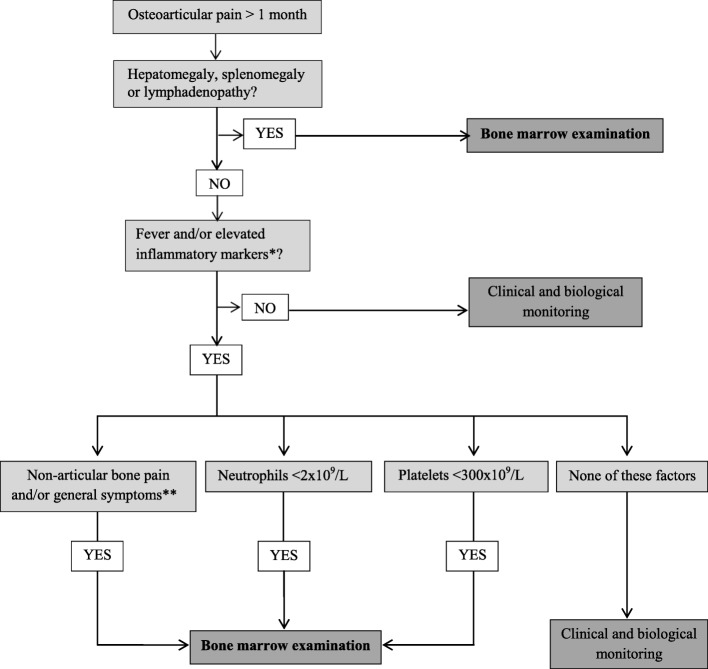
Decision tree proposal for children presenting with at least 1 month of osteoarticular pain. * “Elevated inflammatory markers” were defined as C-reactive protein > 6 mg/L and/or the 1st hour’s erythrocyte sedimentation rate > 20 mm. ** “General symptoms” were defined as the presence of at least one of the following parameters: anorexia, weight loss or asthenia.

Although the time between the onset of symptoms and the start of adapted treatment does not seem to alter the prognosis for children with ALL [11, 25], it was noted that a delay in the diagnosis caused guilt in parents and physicians. In Fig. 1, we propose a decision tree to help clinicians decide when to examine bone marrow in the presence of persistent osteoarticular pain, in order to diagnose ALL as early as possible, before the appearance of hepatomegaly, splenomegaly or lymphadenopathy, high lymphocyte levels, cytopenia and blasts in peripheral blood smear. We reviewed the medical records of nine patients from our study, followed by a rheumatologist and later diagnosed with ALL. The management and diagnosis for these patients could be discussed by using this decision tree. When applying it, their medical records suggest that a bone marrow examination should have been performed earlier for all of them. During their medical follow-up, all had general symptoms (especially asthenia) and/or non-articular bone pain and/or discordance between neutrophils < 2 × 10^9^/L or platelets < 300 × 10^9^/L associated with fever or elevated inflammatory markers. This decision tree could have reduced the time to diagnosis for these nine patients. Their median time to diagnosis was 82 days [IQR: 54–143] compared to 57 days [IQR: 38–90] for the entire group. A future prospective multicenter study with a more substantial sample size, including children followed up for persistent osteoarticular complaints, is necessary in order to confirm these findings and to validate the decision tree.

## Conclusions

In this study, the single most important feature distinguishing ALL from JIA was the presence of hepatomegaly, splenomegaly or lymphadenopathy. If these manifestations are disregarded, bone pain and/or general symptoms (asthenia, anorexia or weight loss), neutrophils < 2 × 10^9^/L, and platelets < 300 × 10^9^/L were associated with the presence of ALL and should prompt a bone marrow examination in the presence of fever or elevated inflammatory markers. Based on our findings, we propose a preliminary decision tree that could be tested in prospective studies.

## Data Availability

The datasets used and/or analyzed during the current study are available from the corresponding author on reasonable request.

## Abbreviations

AIC: Akaike information criterion
ALL: Acute lymphoblastic leukemia
AUC: Area under the ROC curve
CI: Confidence interval
CRP: C-reactive protein
ESR: Erythrocyte sedimentation rate
HCT: Hematocrit
Hgb: Hemoglobin
ILAR: International League of Associations for Rheumatology
IQR: Interquartile range
JIA: Juvenile idiopathic arthritis
MCHC: Mean corpuscular hemoglobin concentration
MCV: Mean corpuscular volume
OR: Odds ratio
WBC: White blood cells
